# Global Learning for Health Equity: A Literature Review

**DOI:** 10.5334/aogh.3810

**Published:** 2022-10-17

**Authors:** Yolanda Ogbolu, Ruth Dudding, Kevin Fiori, Janette North-Kabore, Dana Parke, Rev. Alexander Plum, Sonya Shin, Virginia Rowthorn

**Affiliations:** 1 University of Maryland Baltimore, Center for Health Equity and Outcomes Research, US; 2 Athens City County Health Department, US; 3 Montefiore Medical Center, The University Hospital for Albert Einstein College of Medicine, Department of Family & Social Medicine, US; 4 University of Maryland Baltimore, School of Nursing, US; 5 Henry Ford Health System, Population Health Management, US; 6 Corner Health Center, US; 7 Brigham and Women’s Hospital, Harvard Medical School, US; 8 University of Maryland Baltimore, Center for Global Engagement, US

**Keywords:** global learning, health equity, literature review, health disparities, reverse innovation, frugal innovation, bi-directional learning, reciprocal learning

## Abstract

**Background:**

In high income countries struggling with escalating health care costs and persistent lack of equity, there is growing interest in searching for innovative solutions developed outside national borders, particularly in low- and middle-income countries (LMICs). Engaging with global ideas to apply them to local health equity challenges is becoming increasingly recognized as an approach to shift the health equity landscape in the United States (US) in a significant way. No single name or set of practices yet defines the process of identifying LMIC interventions for adaptation; implementing interventions in high-income countries (HIC) settings; or evaluating the implementation of such projects.

**Objectives:**

This paper presents a review of the literature describing the practice of adapting global ideas for use in the US, particularly in the area of health equity. Specifically, the authors sought to examine; (i) the literature that advocates for, or describes, adaption of health-related innovations from LMICs to HICs, both generally and for health equity specifically, and (ii) implementation practices, strategies, and evidence-based outcomes in this field, generally and in the area of health equity specifically. The authors also propose terminology and a definition to describe the practice.

**Methods:**

The literature search included two main concepts: global learning and health equity (using these and related terms). The search consisted of text-words and database-specific terminology (e.g., MeSH, Emtree) using PubMed, Embase (Elsevier), CINAHL (Ebsco), and Scopus in March 2021. The authors also contacted relevant experts to identify grey literature. Identified sources were categorized according to theme to facilitate analysis. In addition, five key interviews with experts engaged with global ideas to promote health equity in the United States were conducted to develop additional data.

**Results:**

The literature review yielded over ninety (n = 92) sources relating to the adaptation of global ideas from low resource to higher resource settings to promote health equity (and related concepts). Identified sources range from those providing general commentaries about the value of seeking health-related innovations outside the US border to sources describing global projects implemented in the US, most without implementation or outcome measures. Other identified sources provide frameworks or guidance to help identify and/or implement global ideas in the US, and some describe the role of the World Health Organization and other international consortia in promoting a global approach to solving domestic health equity and related challenges.

**Conclusions:**

The literature review demonstrates that there are resources and commentary describing potential benefits of identifying and adapting novel global ideas to address health equity in the US, but there is a dearth of implementation and evaluation data. Terminology is required to define and frame the field. Additional research, particularly in the area of implementation science and evidence-based frameworks to support the practice of what we define as ‘global learning’ for health equity, is necessary to advance the practice.

## Introduction

Increasing recognition of profound health disparities in the United States (US) has stimulated interest in seeking innovative solutions by drawing on experiences beyond this country’s borders. Although the global movement of health care innovations is as old as humankind, the specific call to consider interventions developed in low resource settings to address health care challenges in higher resource countries started gaining steam in the early 2000s [1], when the modern field of global health took off and Global North researchers, particularly in the field of HIV/AIDS, were able to see first-hand the promise of interventions not seen before in the US. Many noted the potential value of low-cost, high-impact innovations to address spiraling health care costs, and to improve primary and preventative health care in the US [2,3,4]. Because many global interventions identified as potentially valuable in US settings derive from countries with universal health care and a focus on primary care, many view opportunities to learn from the world as a strategy to improve health equity in the US.

However, despite strong support in the literature, there has been limited implementation and evaluation data about using this approach, and no consistent agreed upon name for the approach of leveraging global ideas for local impact. The taxonomy used has included the terms; ‘reverse innovation,’ ‘frugal innovation,’ ‘reciprocal innovation,’ and more recently ‘global learning.’ This paper advocates for the use of ‘global learning’ and introduces a proposed definition.

The authors conducted a review of the literature describing the practice of adapting global ideas for use in the US, particularly around health equity. Specifically, the authors sought to examine (i) the literature that advocates for, or describes, adaption of health-related innovations from low-income countries to high income countries, generally and for health equity in specific, and (ii) literature regarding implementation practices, strategies, and evidence-based outcomes in this field generally and for health equity specifically.

### Background: Evolution of ‘global learning’

The term ‘global learning’ was first used in the health context in 1994 by Morgan and Rau in a document that curated dozens of low-income country innovations worthy of adoption in high-income contexts in a process the authors called ‘global learning for health [5].’ However, the terminology did not take hold at that time. Rather, the concept of ‘reverse innovation,’ already a well-established principle in the business world, took hold in the health realm as well [6]. Starting in the early 2000s, there was growing endorsement for reverse innovation as a way to improve health care systems in high-income countries (HICs) [7] and an important pathway to bring needed solutions to countries, particularly the US, struggling with enormous health care costs, inefficiency, and inequity [8]. Recently, the use of the reverse innovation nomenclature has been criticized as limited and potentially derogatory [9].

Often discussed in tandem with reverse innovation is the field of ‘frugal innovation,’ defined by Marco Zeschky et. al., as ‘good-enough, affordable products that meet the needs of resource-constrained consumers [10].’ Escalating demand and global resource constraints are putting pressure on health systems in HICs to deliver more for less, making frugal innovation an attractive option [10].

In much of the literature, no specific terminology for the practice is used. Between 2004 and 2012, a number of papers and reflection pieces were published by HIC authors around the theme of learning from ‘developing’ countries, particularly African countries [2,3,4,11,12,13]. These sources reflected a shift in attitude at the time, linked to the growth of the field of global health, towards partnerships that prioritized mutuality of benefits between countries, including two-way flows of expertise and knowledge [14]. In these articles, health leaders began turning their attention to resource-constrained settings to generate effective and economical solutions for health [14], including Lord Nigel Crisp, the former Chief Executive Officer of the UK National Health Service, who stated, ‘rich countries can learn a great deal about health and health services from poorer ones…combining the learning from rich and poor countries can give us new insight on how to improve health [14].’ At the same time, terms such as bidirectional and reciprocal learning became more common in the literature [15,16].

In 2014, the Robert Wood Johnson Foundation (RWJF) embarked on an effort to actively learn from other countries and surface solutions to accelerate progress towards a Culture of Health in the US. Its ‘Global Ideas for US Solutions’ team began supporting projects that explore how models, policies, and approaches that have helped improve health and well-being abroad could be adapted in the US to advance health equity [17]. As part of this effort, the RWJF team used the term ‘global learning’ to describe the approach. The renewed use of the term ‘global learning’ was strengthened in 2020 when RWJF supported the creation of the Global Learning for Health Equity Network to advance the approach in the United States. With this background in mind, the authors recommend the use of the nomenclature ‘global learning’ to describe the field going forward.

In 2018, a significant initiative was launched to highlight the role that global learning could play to advance health equity. The Arnhold Institute for Global Health-led ‘Task Force on Global Advantage’ issued a report that identified a set of global approaches that could ‘yield breakthroughs in the health of America’s most vulnerable communities [18].’ The report focused on an analysis of three countries (Brazil, Rwanda, Ethiopia) that have significantly improved health outcomes by making primary care accessible in communities. Equity was one of the primary concepts embraced in the report’s findings and recommendations. The report further noted that, at the heart of global learning is diffusion, dissemination, and the implementation of ideas.

An additional thread in the growing recognition of global learning as a valuable health equity strategy is the current work of the World Health Organization (WHO), United Nations (UN), and other international bodies to encourage action on the social determinants of health (SDoH) to reduce inequities in health [19]. The role of the WHO in leading international efforts to address health equity was strongly enunciated in the WHO report, ‘Closing the gap in a generation: health equity through action on the social determinants of health [20].’ The report does not reference reverse innovation or global learning specifically, but clearly advocates for it in one of the report’s recommendations:

*Generating and disseminating social determinants of health knowledge*: Ensure research funding is allocated to social determinants of health work; support the global health observatory and multilateral, national, and local cross-sectoral working through development and testing of social determinants of health indicators and intervention impact evaluation; establish and expand virtual networks and clearing houses organized on the principles of open access, managed to enhance accessibility from sites in all high-, middle-, and low-income settings; … [20(p33)]

The most recent manifestation of WHO’s advocacy around the SDoH approach to health equity is an initiative to develop a global framework and basket of core indicators to monitor progress on key SDoH-focused actions of governments to improve health equity [21].

The aims of this literature review were to 1) examine nomenclature used to discuss approach of using global ideas to advance health equity, 2) identify current research and programs engaged in global learning, and 3) provide insights and recommendations for future action to advance the field of global learning.

## Methods

The literature review focused on two main concepts: global learning and health equity (using these and related terms). The authors compiled sources in two stages: 1) a search of peer-reviewed literature, and 2) outreach to experts to identify additional academic literature as well as grey literature such as commentaries, news items, and reports from think-tanks. In the first phase of this review, relevant academic literature was identified using text-words and database-specific terminology (e.g., MeSH, Emtree). The authors did not restrict results by date. The search was conducted using PubMed, Embase (Elsevier), CINAHL (Ebsco), and Scopus in March 2021. The authors used Covidence, a systematic review software, to manage references, remove duplicates, and conduct title abstract screening to determine relevance to the inclusion and exclusion criteria. Following the abstract review, the reviewers screened the full text of potentially relevant papers independently to determine if they met inclusion criteria for this review.

### Expert Interviews

In addition to the literature review, the authors recruited five experts and conducted five semi-structured interviews to complement and contextualize findings from the literature review. Using a combined approach is especially useful when the state of the science is nascent and further context could help to better understand current structures, gaps, and opportunities for development. Expert interviews were conducted using a semi-structured interview guide that addressed topics such as personal experience with globally sourced interventions; evidence for using a global approach in general and to advance health equity specifically; knowledge of implementation strategies, evidence-based principles, and evaluation strategies related to this approach; and recommendations for advancement of the field. The authors defined experts as individuals who have engaged with global ideas, either in academia or in practice. All interviews were conducted through one-hour video conference calls between March to June 2021. Interviews were analyzed for key themes.

## Results

**Figure 1:**
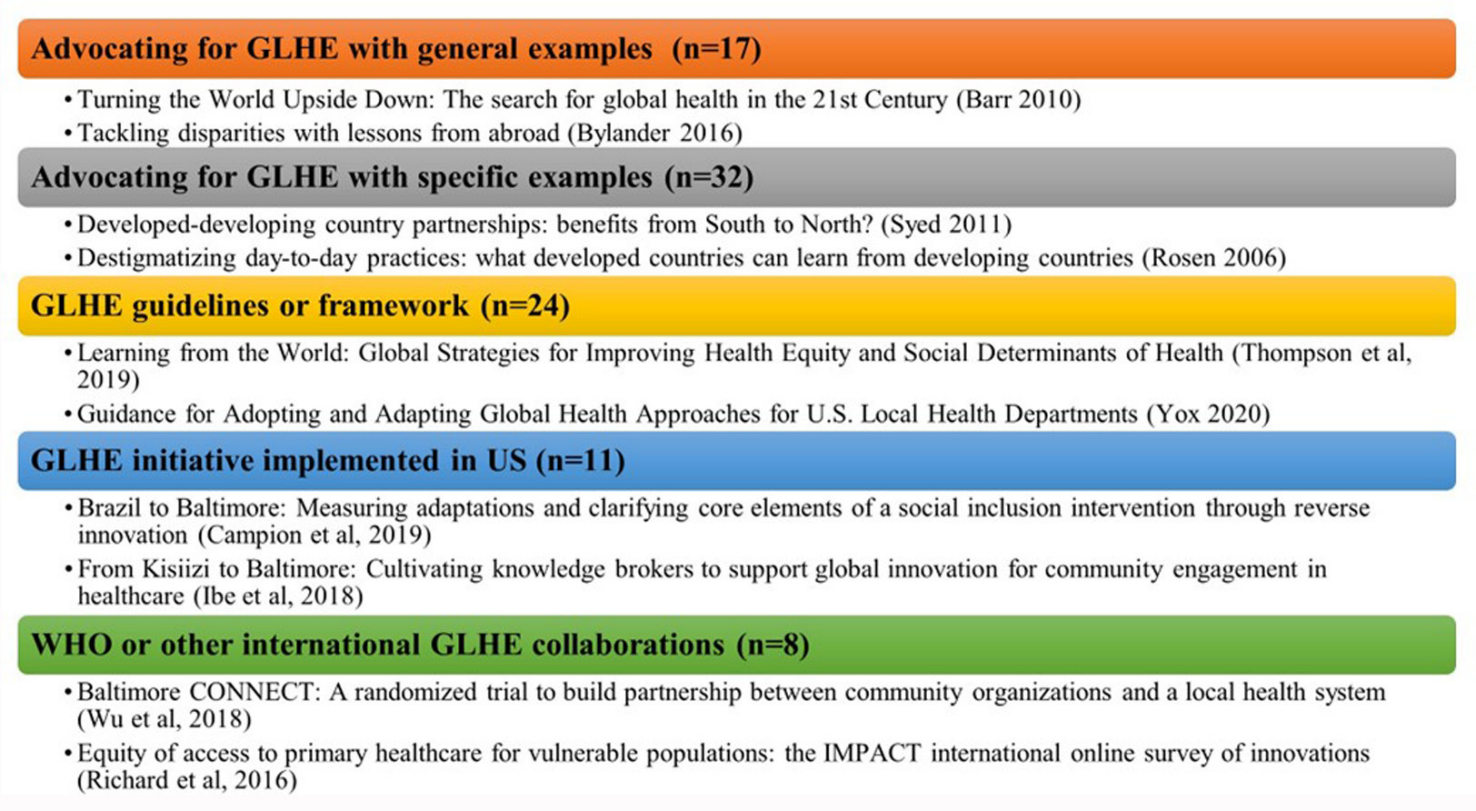
Literature review categories with examples.

The literature search returned a total of 677 potential sources. After removing duplicates, 470 papers underwent abstract screening for relevance. Of these, 164 papers were identified as potentially relevant and underwent complete review by two authors. After consultation and discussion between both authors, 73 papers were excluded and 92 were determined by both authors as meeting eligibility criteria for this review. The included sources were grouped into categories co-developed by the authors to facilitate analysis. The categories (see Figure 1) identified sources along a continuum from general to specific based on the degree to which the document described global learning generally or referenced specific interventions.

### Advocating for global learning with general examples

Seventeen (17) sources [2,3,4,8,11,12,13,22,30,31,32,33,34,35,36,37,38] were categorized by the authors as broadly describing the potential benefits of learning from low-income or low-resource settings; [12] the developing world, developing countries, or developing nations; [4,13] Africa; [2] or abroad [11]. The sources in this category do not provide details about specific interventions, but rather comment on the broader financing and equity justifications for global learning.

The sources in this category do not use the term ‘health equity,’ but reference the potential for global learning to improve health and wellbeing [30], reduce the burden of disease on vulnerable populations [36,37], and tackle health-related disparities [11]. Overwhelmingly, the sources promote global learning as a way to access low-cost, high-impact, and innovative health care and public health interventions developed out of necessity in low resource settings. A sample statement is as follows:

In constrained environments, where resources are scarce, healthcare providers often craft unexpected solutions to provide adequate healthcare to patients. These inexpensive but effective frugal innovations may be imperfect, but they have the power to improve people’s lives by ensuring that health is within everyone’s reach [12(p.3)].

Many of the sources in this general category cite a lack of evidence-based practices and the need for a better understanding of why some initiatives flourish in the US while others do not.

### Advocating for global learning with specific examples

Thirty-two (32) sources [4,14,23,39,40,41,42,43,44,45,46,47,48,49,50,51,52,53,54,55,56,57,58,59,60,61,62,63,64,65,66,67] were categorized as advocating for global learning with specific examples of initiatives to promote health equity in the US (or a term related to health equity). These sources promote the benefits of global learning and additionally present specific examples of initiatives developed outside the US that could either address a particular challenge in the US health care system or improve the overall functioning of the US health care system. Examples of the former describe initiatives to address mental health disparities [42,63], promote person-centered care [46], and address the COVID-19 pandemic [64,67], non-communicable diseases [50], patient safety [15], and cardiac care [61]. Examples of the latter describe global learning as a way to stimulate health care innovation [41,55,58,68] and as an approach to cost-effective health care [57].

### Global learning guidelines or frameworks

Twenty-four (24) sources [5,6,7,9,16,18,24,25,68,69,70,71,72,73,74,75,76,77,78,79,80,81,82,83] provide guidance, checklists, or frameworks to support or overcome barriers to global learning. Four of the included frameworks are specifically designed to promote health equity:

- Learning From Others: Comprehensive Health Equity Strategies in Europe [76]
- Shared Learning in an Interconnected World: Innovations to Advance Global Health Equity [69]
- Learning from the World: Global Strategies for Improving Health Equity and Social Determinants of Health [24]
- Impact Innovation: Closing Health Inequities Through Design [77]

Sources in this category include relatively simple guidelines and frameworks based on the authors’ observations as global health researchers; frameworks developed by experts within organizations interested in or practicing global learning; and frameworks developed and refined through systematic research and/or expert panels. Some of the papers in this category highlighted barriers and facilitators to global learning including legal and regulatory barriers and recommendations for overcoming them [81]. One of the papers also identified challenges, resistance, and facilitators that should be considered for uptake of global learning [16]. Challenges and resistance included prejudice to the reverse innovation process, doubt that something could be learned from a developing country, and concerns related to cultural differences. Facilitators were described as bi-directional engagement of leaders and communication of the financial and quality care benefits of innovations.

### WHO or other international consortia

Eight (8) sources [15,20,28,84,85,86,87,88] were included that describe a health equity initiative implemented in multiple sites by the WHO or another international collaborative. International multi-site implementation initiatives are not new, and the search terms certainly did not capture the breadth of this practice. Some sources in this category describe initiatives first developed in an LMIC that were later implemented in multiple settings via an international consortium. For instance, one of the sources describes the African Partnerships for Patient Safety (APPS) program that created hospital partnerships using a ‘learn and do’ approach in six African countries and partner hospitals in England and Switzerland to co-develop tools and resources [15]. Other sources in this category promote an international collective approach to global learning where the best ideas and practices are developed, shared, and/or tested in multiple sites.

### Global learning initiatives implemented in the US

As depicted in Figure 1, few published sources (N=11) [16,26,27,89,90,91,92,93,94,95,96] report actual implementation of global ideas into local US communities to promote health equity or a related outcome. Most sources in this category are brief overviews of projects, and very few describe the full implementation and associated health equity outcomes. Mendel et al. is an exception in that it details a completed study examining the acceptability and feasibility outcomes of the adaptation of experience-based co-design (EBCD) from six countries to Los Angeles County [89]. Many of the papers in this category discuss process outcomes rather than health equity outcomes. Process outcomes included the development of community engagement strategies and knowledge brokerage approaches [28,90], strategy papers and policy briefs [91], conceptual frameworks [92], training outcomes for community health workers [93], and implementation science outcome measures [26].

A range of health equity issues and outcomes were described within this subset of papers. Outcomes included increased social participation in health [92]; HIV access and treatment outcomes [95]; health care disparities, and social participation for underrepresented groups [89,96]; community engagement outcomes [27]; community resilience and, wellbeing [90]; mental health [89]; and social isolation [26].

#### Expert Interviews

Five (5) key stakeholders in the global learning field were interviewed. They detailed global learning occurring in a variety of institution types including the US government, non-profit organizations, an academic institution, and a research institution. Similar themes emerged from the interviews. First, all agreed that the field of global learning is nascent and evolving and requires a unifying name.

The experts also indicated that the concept of global learning should include elements that are not always included in discussions of reverse innovation, specifically bidirectional learning and community engagement. Both concepts reflect the belief that equitable processes are more likely to lead to equitable outcomes and the importance of moving away from a hierarchical and potentially colonizing process of knowledge extraction and transfer. Experts concurred that global learning must not be a new form of colonization where ideas are taken without acknowledgement of the originating site.

Second, the experts also agreed that, in practice, engagement with global ideas takes many forms, from inspiration to high fidelity adaptation of a global idea, and warned that supporting a strict conception of the practice can make it harder to engage with communities and donors who want a variety of approaches. Finally, the experts enunciated the need for support for global learning in terms of funding, tools, best practices, and networks to encourage uptake of the approach.

Several experts offered recommendations for expanding the identification of global innovations and reducing the barriers to engaging in global learning. One expert concluded that the main challenge to the growth of global learning is not the sourcing of good global ideas or innovations, but the lack of market demand in the US. He noted that people are less likely to see relevance in unfamiliar sources, perceiving, among other things, that a greater effort would be required to incorporate novel solutions, which he described as classic diffusion dilemma. He recommended stimulating diffusion and demand by providing more organizational support and advancing dissemination of knowledge through the development of social and professional networks of organizations.

## Discussion and Recommendations

The literature review and interviews suggest that seeking novel approaches outside of our borders is a promising approach to advance health equity in the US, but it is an approach that is not well-defined, not frequently used, and still lacks implementation strategies and evaluation measures. The literature review identified twenty-four guidelines or frameworks for global learning that take a multitude of different approaches. To move the field forward, stakeholders should consider whether a new framework is required or whether elements of existing frameworks or models can be combined or refined to support adaptation of global ideas to advance health equity in the US.

Based on the findings of the literature review and interviews, the authors suggest the following recommendations:

1. Unifying nomenclature and definition required

The approach of engaging with, identifying, adapting, and evaluating global ideas from low resource settings to higher resource settings should be consolidated under a unifying term and definition, especially given that the prior organizing term—reverse innovation—has gone out of favor given its inappropriate implication that the natural course of innovation is from HICs to LMICs. The term ‘global learning,’ first introduced in 1994, is resurging and starting to gain traction among scholars or practitioners. As such, the authors recommend the use of the term ‘global learning’ to describe the field.

In terms of fleshing out the definition of global learning, particularly in the area of health equity, several expert interviewees stated that their understanding of global learning adapted over time, moving from a narrow focus on identifying and adapting the core elements of an intervention from an LMIC to an HIC to a broader definition that reflects global learning as a spectrum of activities. One paper set forth four ways global learning can be used in HICs:

- adoption of a specific intervention developed in another country, maintaining core elements from the originator site
- an orientation to inform development of local ideas with global ideas
- adaptation of microelements of the original initiative
- joint learning and problem solving with global communities facing similar challenges [97].

Also, several interviewees and included sources referenced that global learning happens when individuals from multiple settings come together to co-develop [41] or multisolve innovations [88]. Notwithstanding how global learning emerges, many cautioned that global learning must not be extractive or appropriative of the investment and expertise of the originating community.

With this input, the authors determined that a broad definition of global learning is preferable to address the difficulty identified by key interviewees of trying to operationalize a narrow concept of global learning. Organizations interested in global learning will likely have to adopt multiple approaches and have the flexibility to respond to multiple community needs. Another area of flexibility emerging from expert interviews and literature review was the value of a definition that supports sharing between all countries—not just from an LMIC to a HIC. The field of global learning is built on the important notion that higher resource settings can and should learn from low resourced settings and appreciate the critical knowledge generated by historically under-valued communities. However, in the effort to advance health equity, all sources and settings must be considered. With this background, the authors propose the following terminology and definition for global learning for health equity purposes:

*Global learning for health equity is the practice of engaging with, exchanging, and adapting health equity-promoting ideas and interventions between communities in ways that foster implementation benefits that are reciprocal and beneficial to both*.

2. Concrete support and resources are needed for global learning for health equity

The practice of global learning is supported by prominent advocates in global health and public health fields both in and outside the US, as well as some of the most prominent health-focused organizations in the US including National Institutes of Health (NIH), National Association of County and City Health Officials (NAACHO), and the Institute for Health Care Improvement. This support should be leveraged to increase demand for global learning in the US. Evidence elicited from experts and documentary sources noted the critical need for funding to support global learning initiatives and pilot projects in the US, as well as a concerted effort to raise awareness about the importance of global learning and increase market demand. Identification of potential translatable interventions was not raised as a barrier to uptake of global learning given the existence of a number of registries and databanks of global innovations.

3. Project-specific implementation and outcomes research is necessary to progress the field of global learning for health equity

Although considered a promising approach, global learning is challenging, and the lack of empirically grounded examples of global learning indicates that there is still much work to be done in understanding and promoting this type of innovation flow. To date, international application of diffusion, dissemination, and implementation concepts has either focused on diffusion of an HIC innovation to LMICs or on tracing the spread of an innovation across many countries [98]. Scholars have asked whether the same factors are important when the innovation moves from an LMIC to an HIC [98]. Understanding, facilitating, evaluating all aspects of knowledge transfer as it relates to global learning is critical to promoting the approach.

The need for additional research was a consistent theme in the literature review and across all expert interviews. One source set forth areas of outstanding research needed to bolster global learning:

There is a need for studies that explore the challenges around *(1)* innovation identification; *(2)* necessary specificities of the adopter site; *(3)* partnerships and the key success factors to persuade and galvanize support; *(4)* testing for safety and effectiveness; *(5)* adaptation strategies to fit local contexts; and *(6)* economic evaluations to understand where savings can be usefully spread throughout the system [83(pp26–30)].

Implementation science research is required to develop a clear picture about what initiatives are adaptable, what aspects of the intervention can be adapted, defining and understanding the value of ‘core components,’ and when extensive adaptation creates a new intervention altogether [78].

4. The World Health Organization and international consortia should play a stronger role in global learning

International organizations such as the WHO have a critical role in disseminating evidence-based innovations from around the world. Several documents included in the literature review describe initiatives spearheaded by the WHO or another international collaborative, and this role was encouraged by the expert interviewees. One included source identified that ‘the World Health Organization has a particular role in providing guidance and promulgating knowledge [in global learning] [47].’ Another described the WHO African Partnerships for Patient Safety (APPS) program that was launched in 2008 in response to a WHO technical report on patient safety issues and solutions in African health systems and twelve action areas [15]. Since that time, APPS has grown through establishment of mutually beneficial hospital partnerships co-developed by frontline health workers in Africa. As the project progressed, the value of bidirectional learning was established: ‘improvement in African hospitals had clear relevance to improving capacity in partner hospitals in Europe [15].’

Some have argued that the WHO has, at times, perpetuated a one-way flow of innovation by, for instance, promoting technologies in LMICs brought in from HICs and making no attempt to enlist the participation of local populations in planning or implementation [99]. This further illustrates the importance of ensuring that global learning is not exploitive but rather embodies principles of community engagement and mutually beneficial relationships.

Yet another source described aspects of the IMPACT (Innovative Models Promoting Access-to-Care Transformation) study, a 5-year Canadian-Australian research program aiming to identify, implement, and trial best practice interventions to improve access to primary health care for vulnerable populations [84]. Although primarily focused on sharing strategies between two HICs, the document noted the value of this kind of consortia-based work to encourage sharing of strategies for vulnerable populations in different global settings [85].

The nascent global learning movement in the US dovetails with the global undertaking led by the WHO, UN, and other international bodies to encourage action on the SDoH to reduce inequities in health. This effort on the part of the WHO includes development of successful policies and implementation plans and identification and sharing of best practices across the globe: all of which are key features of the global learning. The role of international health organizations in global learning is a critical area of future study and action.

## Conclusion

If this literature review had limited its search terms to ‘global learning for health equity,’ no sources would have met the inclusion criteria. This reflects the youth of the field. However, several factors are converging that will propel global learning for health equity forward, all of which have been amplified by the global impact of the COVID-19 pandemic and racial reckoning movement in the US. The dual crises illuminated challenges with health inequities and the inadequacies of the US health care system. The international pandemic response demonstrated multiple national approaches to a single challenge, highlighting the breadth of innovation available to the US if we are open to new ideas coming from outside our borders. The global learning movement fills a critical need by supporting the identification of low-cost, high-impact interventions that hold the promise of advancing health equity in the US.

## Limitations

The goal of this literature review is to provide an overview of the available evidence on global learning for health equity, a topic that is fairly new. The review is helpful for clarifying key concepts and definitions in the literature but has some limitations. While global ideas have no borders, the authors focused on innovations developed in LMICs and implemented in HICs. This is a limitation, and future reviews could expand to include global ideas from all settings.

Another limitation is that some articles may have been missed due to inconsistency in terminology used to describe global learning. Even when global learning terminology was utilized, it may not have been associated with health equity. The search was broad initially, with the number of articles reaching zero when limited to the words ‘global learning for health equity.’ Most articles were descriptive and did not completely describe the full implementation of global learning projects and their associated outcomes. The goal of this review was not to evaluate the quality of the evidence and the information gathered revealed a wide range of study designs and methods. These limitations confirm that the field of global learning for health equity is young and emerging.

## References

[1] Holst J. Global health—emergence, hegemonic trends and biomedical reductionism. Global Health. 2020; 16(1): 42. DOI: 10.1186/s12992-020-00573-4 32375801 PMC7201392

[2] Murray SA. Out of Africa: some lessons for general practice/family medicine in developed countries? Fam Pract. 2000; 17(5): 361–363. DOI: 10.1093/fampra/17.5.361 11021892

[3] Richards T, Tumwine J. Poor countries make the best teachers: Discuss: It is not only what you spend on health but how you spend it. BMJ: British Medical Journal. 2004; 329(7475): 1113–1114. http://www.jstor.org.proxy-hs.researchport.umd.edu/stable/25469396. DOI: 10.1136/bmj.329.7475.1113 15539642 PMC527670

[4] Berwick DM. Lessons from developing nations on improving health care. BMJ. 2004; 328(7448): 1124–1129. DOI: 10.1136/bmj.328.7448.1124 15130984 PMC406330

[5] Harris M, Weisberger E, Silver D, Dadwal V, Macinko J. That’s not how the learning works—the paradox of reverse innovation: a qualitative study. Global Health. 2016; 12(1): 36. DOI: 10.1186/s12992-016-0175-7 27381466 PMC4932777

[6] Harris M, Weisberger E, Silver D, Macinko J. They hear ‘Africa’ and they think that there can’t be any good services—perceived context in cross-national learning: a qualitative study of the barriers to reverse innovation. Global Health. 2015; 11(1): 45. DOI: 10.1186/s12992-015-0130-z 26582041 PMC4652406

[7] DePasse JW, Lee PT. A model for ‘reverse innovation’ in health care. Global Health. 2013; 9(1): 40. DOI: 10.1186/1744-8603-9-40 24001367 PMC3844499

[8] Harris M, Dadwal V, Wu A, Syed SB. It takes threat of Ebola to see lessons from low-income countries. Global Health. 2015; 11(1): 16. DOI: 10.1186/s12992-015-0102-3 25880681 PMC4408579

[9] Lokugamage AU, Douglass C, Gishen F, Fyfe MV. Reverse innovation, power imbalances, language, and avoiding cultural appropriation. BMJ. Published online December 17, 2019; l7003. DOI: 10.1136/bmj.l7003 31848136

[10] Zeschky M, Widenmayer B, Gassmann O. Frugal innovation in emerging markets. Research-Technology Management. 2011; 54(4). DOI: 10.5437/08956308X5404007

[11] Bylander J. Tackling disparities with lessons from abroad. Health Aff. 2016; 35(8): 1348–1350. DOI: 10.1377/hlthaff.2016.0794 27503956

[12] Tran VT, Ravaud P. Frugal innovation in medicine for low resource settings. BMC Med. 2016; 14(1): 102. DOI: 10.1186/s12916-016-0651-1 27383644 PMC4936019

[13] Dockser MA. To fix health care, some study developing world. The Wall Street Journal. Published July 2, 2009. Accessed January 24, 2022. https://www.wsj.com/articles/SB124648865046182847.

[14] Syed S, Dadwal V, Pittet D. Developed-developing country partnerships: benefits from South to North? BMC Proc. 2011; 5(S6): O23. DOI: 10.1186/1753-6561-5-S6-O23

[15] Syed SB, Syed SB, Gooden R, et al. African partnerships for patient safety: a vehicle for enhancing patient safety across two continents. [corrected]. World Hosp Health Serv. 2009; 45(4): 24–27. 20411829

[16] Johnson CD, Noyes J, Haines A, et al. Learning from the Brazilian Community Health Worker Model in North Wales. Global Health. 2013; 9(1): 25. DOI: 10.1186/1744-8603-9-25 23764067 PMC3681592

[17] Robert Wood Johnson Foundation. Global learning: Ideas to improve health in the US—RWJF. Published 2021. Accessed February 1, 2022. https://www.rwjf.org/en/how-we-work/grants-explorer/featured-programs/global-learning.html.

[18] Adam C, Kaosar Afsana W, Sonia Angell B, et al. The Task Force on Global Advantage report; 2018. Accessed January 26, 2022. https://icahn.mssm.edu/files/ISMMS/Assets/Research/Arnhold/TheTaskForceonGlobalAdvantageReport.pdf.

[19] Marmot M, Friel S, Bell R, Houweling TA, Taylor S. Closing the gap in a generation: health equity through action on the social determinants of health. The Lancet. 2008; 372(9650): 1661–1669. DOI: 10.1016/S0140-6736(08)61690-6 18994664

[20] WHO Commission on Social Determinants of Health. Closing the gap in a generation: Health equity through action on the social determinants of health. Final report of the Commission on Social Determinants of Health. World Health Organization; 2008.

[21] World Health Organization. Global monitoring of action on the social determinants of health: a proposed framework and basket of core indicators. Published online 2016. Accessed February 6, 2022. http://www.who.int/social_determinants/monitoring-consultation/en/.

[22] Barr H. Turning the world upside down: The search for global health in the 21st Century, Nigel Crisp, London: Hodder Education 2010. J Interprof Care. 2011; 25(5): 386–387. DOI: 10.3109/13561820.2011.590050

[23] Rosen A. Destigmatizing day-to-day practices: what developed countries can learn from developing countries. World Psychiatry. 2006; 5(1): 21–24. https://pubmed.ncbi.nlm.nih.gov/16757986. 16757986 PMC1472257

[24] Thompson G, Lenzi R, Phillips T, et al. Learning from the World Global Strategies for Improving Health Equity and Social Determinants of Health; 2019. Accessed January 24, 2022. https://www.fhi360.org/resource/learning-world-global-strategies-improving-health-equity-and-social-determinants-health.

[25] Yox E. Guidance for adopting and adapting global health approaches for US local health departments. Published online 2020.

[26] Campion Dialo N, Ogbolu Y, Cordeira V, Velloso C, Esteves G. Brazil to Baltimore: Measuring adaptations and clarifying core elements of a social inclusion intervention through reverse innovation. In: 12th Annual Conference on the Science of Dissemination and Implementation in Health; 2019. https://academyhealth.confex.com/academyhealth/2019di/meetingapp.cgi/Paper/36519.

[27] Ibe CA, Basu L, Gooden R, et al. From Kisiizi to Baltimore: cultivating knowledge brokers to support global innovation for community engagement in healthcare. Global Health. 2018; 14(1): 19. DOI: 10.1186/s12992-018-0339-8 29426345 PMC5807858

[28] Wu AW, Hwang S, Weston CM, et al. Baltimore CONNECT: A randomized trial to build partnership between community organizations and a local health system. Prog Community Health Partnersh. 2018; 12(3): 297–306. DOI: 10.1353/cpr.2018.0054 30581173

[29] Richard L, Furler J, Densley K, et al. Equity of access to primary healthcare for vulnerable populations: the IMPACT international online survey of innovations. Int J Equity Health. 2016; 15(1): 64. DOI: 10.1186/s12939-016-0351-7 27068028 PMC4828803

[30] Zinsstag J, Pelikan K, Hammel T, et al. Reverse innovation in global health. J Public Health Emerg. 2019; 3: 2–2. DOI: 10.21037/jphe.2018.12.05

[31] Vanderbilt T. Reverse innovation’ could save lives. Why aren’t we embracing it? The New Yorker. Published online February 2019.

[32] Yox E. Lessons from the world: Applying global thinking to local public health. Published online August 2019. Accessed January 24, 2022. https://regroup-production.s3.amazonaws.com/documents/ReviewReference/315205652/Yox%20-%202019%20-%20Lessons%20from%20the%20World%20Applying%20Global%20Thinking%20to%20Local%20Public%20Health-annotated.pdf?AWSAccessKeyId=AKIAJBZQODCMKJA4H7DA&Expires=1643146783&Signature=%2F97FUqYipqWHnkHB9sTwIa5ehCE%3D.

[33] Syed SB, Dadwal V, Martin G. Reverse innovation in global health systems: towards global innovation flow. Global Health. 2013; 9(1): 36. DOI: 10.1186/1744-8603-9-36 23992598 PMC3846449

[34] Thunhurst CP. Public health systems analysis—where the River Kabul meets the River Indus. Global Health. 2013; 9(1): 39. DOI: 10.1186/1744-8603-9-39 24119439 PMC3766021

[35] Susser E, Collins P, Schanzer B, Varma VK, Gittelman M. Topics for our times: can we learn from the care of persons with mental illness in developing countries? Am J Public Health. 1996; 86(7): 926–928. DOI: 10.2105/AJPH.86.7.926 8669514 PMC1380431

[36] Swartz HA, Rollman BL. Managing the global burden of depression: lessons from the developing world. World Psychiatry. 2003; 2(3): 162–163. https://pubmed.ncbi.nlm.nih.gov/16946927. 16946927 PMC1525095

[37] Uwemedimo OT, Arora G, Russ CM. New views on global child health: global solutions for care of vulnerable children in the United States. Curr Opin Pediatr. 2016; 28(5): 667–672. DOI: 10.1097/MOP.0000000000000402 27434718

[38] Reverse innovation in global health systems: learning from low-income countries. BMC. Accessed May 24, 2022. https://www.biomedcentral.com/collections/reverseinnovations.

[39] Caine J, Pokhrel K. Stories from the field. Health Promot Pract. 2011; 12(6_suppl_2): 199S–206S. DOI: 10.1177/1524839911419295 22068583

[40] Benatar S, Sullivan T, Brown A. Why equity in health and in access to health care are elusive: Insights from Canada and South Africa. Glob Public Health. 2018; 13(11): 1533–1557. DOI: 10.1080/17441692.2017.1407813 29202651

[41] Crisp N. Co-development, innovation and mutual learning—or how we need to turn the world upside down. Healthcare. 2015; 3(4): 221–224. DOI: 10.1016/j.hjdsi.2015.06.002 26699347

[42] Walters AS. The friendship bench: Delivering mental health treatment in developing countries. The Brown University Child and Adolescent Behavior Letter. 2020; 36(4): 8–8. DOI: 10.1002/cbl.30457

[43] Bewes P. Learning from low-income countries: what are the lessons? Trained medical assistants can successfully do work of doctors. BMJ. 2004; 329(7475): 1184.2. DOI: 10.1136/bmj.329.7475.1184-a PMC527736 15539688

[44] Rabkin M, El-Sadr WM, Mugyenyi P, Ramatlapeng MK, de Cock KM. Lessons from Africa. JAIDS Journal of Acquired Immune Deficiency Syndromes. 2010; 55(Supplement 2): S141–S143. DOI: 10.1097/QAI.0b013e3181fbcb76 21406985 PMC3561635

[45] Basu L, Pronovost P, Molello NE, Syed SB, Wu AW. The role of South-North partnerships in promoting shared learning and knowledge transfer. Global Health. 2017; 13(1): 64. DOI: 10.1186/s12992-017-0289-6 28830489 PMC5568264

[46] Basu L, Frescas R, Kiwelu H. Patient guardians as an instrument for person centered care. Global Health. 2014; 10(1): 33. DOI: 10.1186/1744-8603-10-33 24885655 PMC4022410

[47] Crisp N. Mutual learning and reverse innovation–where next? Global Health. 2014; 10(1): 14. DOI: 10.1186/1744-8603-10-14 24673828 PMC3986825

[48] Bhatti Y, Taylor A, Harris M, et al. Global lessons in frugal innovation to improve health care delivery in the United States. Health Aff. 2017; 36(11): 1912–1919. DOI: 10.1377/hlthaff.2017.0480 29137503

[49] Arshad H, Radić M, Radić D. Patterns of Frugal Innovation in Healthcare. Technology Innovation Management Review. 2018; 8(4): 28–37. DOI: 10.22215/timreview/1150

[50] Bollyky TJ. New, cheap, and improved: Assessing the promise of reverse and frugal innovation to address noncommunicable diseases. Council on Foreign Relations.

[51] Matheson A, Bourke C, Verhoeven A, et al. Lowering hospital walls to achieve health equity. BMJ. Published online September 20, 2018; k3597. DOI: 10.1136/bmj.k3597 30237307 PMC6146487

[52] Roerty S. How can urban planning contribute to building health equity? British Medical Journal. Published April 16, 2020. Accessed January 24, 2022. https://blogs.bmj.com/bmj/2020/04/16/how-can-urban-planning-contribute-to-building-health-equity/.

[53] King A, Odunitan-Wayas F, Chaudhury M, et al. Community-based approaches to reducing health inequities and fostering environmental justice through global youth-engaged citizen science. Int J Environ Res Public Health. 2021; 18(3): 892. DOI: 10.3390/ijerph18030892 33494135 PMC7908382

[54] National Academies of Sciences E and M. Communities in action: Pathways to health equity; 2017. Accessed January 24, 2022. https://nam.edu/programs/culture-of-health/communities-in-action-pathways-to-health-equity/?gclid=Cj0KCQjw9YWDBhDyARIsADt6sGbRzdaXWJNoC4l-TT1EtT-GaHDzTsdMBq76lSAuzHd23rUZbeOT4KEaAo0fEALw_wcB. 28418632

[55] Darzi A, Parston G, Harris M, Bhatti Y, Prime M, del Castillo J. Global diffusion of healthcare innovation making the connections; 2016. Accessed January 24, 2022. https://www.wish.org.qa/reports/.

[56] Kouri D. Learning from others: Health equity strategies and initiatives from Canadian regional health authorities; 2013. Accessed January 24, 2022. https://www.wellesleyinstitute.com/publications/learning-from-others-health-equity-strategies-and-initiatives-from-canadian-regional-health-authorities/.

[57] Skopec M, Issa H, Harris M. Delivering cost effective healthcare through reverse innovation. BMJ. Published online November 14, 2019; l6205. DOI: 10.1136/bmj.l6205 31727640

[58] Dandonoli P. Open innovation as a new paradigm for global collaborations in health. Global Health. 2013; 9(1): 41. DOI: 10.1186/1744-8603-9-41 24000780 PMC3847159

[59] Cotton M, Henry J, Hasek L. Value innovation: an important aspect of global surgical care. Global Health. 2014; 10(1): 1. DOI: 10.1186/1744-8603-10-1 24393237 PMC3892040

[60] Bodeker G. Lessons on integration from the developing world’s experience Commentary: Challenges in using traditional systems of medicine. BMJ. 2001; 322(7279): 164–167. DOI: 10.1136/bmj.322.7279.164 11159579 PMC1119421

[61] Richman BD, Udayakumar K, Mitchell W, Schulman KA. Lessons from India in organizational innovation: A tale of two heart hospitals. Health Aff. 2008; 27(5): 1260–1270. DOI: 10.1377/hlthaff.27.5.1260 18780909

[62] Reid A, Nariño S, Magge H, Sassi A. Advancing equity in health systems by addressing racial justice. Published online 2019. DOI: 10.48558/PF55-BJ56

[63] Potash S. Low-cost mental health innovation enables access to care in Africa, US. Fogarty International Center @ NIH. Published 2018. Accessed January 25, 2022. https://www.fic.nih.gov/News/GlobalHealthMatters/march-april-2018/Pages/low-cost-mental-health-innovation.aspx.

[64] Holmes CB, Goosby EP. How lessons from global health can improve health and the response to COVID-19 in the US. Health Affairs. Published August 10, 2020. Accessed January 25, 2022. https://www.healthaffairs.org/do/10.1377/forefront.20200806.949101/full/.

[65] Hiatt H, Kenney C, Rosenberg M. Global health at home: Harvesting innovation from around the world to improve American medical care. Harv Mag. Published online October 2016.

[66] Furin JJ, Behforouz HL, Shin SS, et al. Expanding global HIV treatment. Ann N Y Acad Sci. 2008; 1136(1): 12–20. DOI: 10.1196/annals.1425.004 17954668

[67] Acharya K. How to see what the world is teaching us about COVID-19. Stanford Social Innovation Review. Published online August 20, 2020. DOI: 10.48558/TKDY-6998

[68] Onie R, Farmer P, Behforouz H. Realigning health with care. Stanford Social Innovation Review. Published online 2012: 28–35.

[69] Binagwaho A, Nutt CT, Mutabazi V, et al. Shared learning in an interconnected world: innovations to advance global health equity. Global Health. 2013; 9(1): 37. DOI: 10.1186/1744-8603-9-37 24119388 PMC3765795

[70] Rowthorn V, Edwards LA, Lipscomb J, Olsen J. Global to local: Methods and models. Ann Glob Health. 2017; 82(6): 951. DOI: 10.1016/j.aogh.2016.11.009 28314496

[71] Snowdon AW, Bassi H, Scarffe AD, Smith AD. Reverse innovation: an opportunity for strengthening health systems. Global Health. 2015; 11(1): 2. DOI: 10.1186/s12992-015-0088-x 25889986 PMC4328056

[72] Sawin E. The magic of ‘multisolving.’ Stanford Social Innovation Review. Published online July 16, 2018. Accessed January 24, 2022. https://ssir.org/articles/entry/the_magic_of_multisolving#.

[73] Chabikuli O, Mastro T. What can the United States learn from other countries to tackle disparities? https://degrees.fhi360.org/. Published October 7, 2020. Accessed January 24, 2022. https://degrees.fhi360.org/2020/10/what-can-the-united-states-learn-from-other-countries-to-tackle-disparities/.

[74] Dearing JW, Lapinski M, Shin SY, et al. A model for introducing global ideas to the US; 2019. Accessed January 24, 2022. https://regroup-production.s3.amazonaws.com/documents/ReviewReference/315205800/Dearing_et_al_2019b.pdf?AWSAccessKeyId=AKIAJBZQODCMKJA4H7DA&Expires=1643171293&Signature=gXGb6QpcRg85%2BMv9hOMtjROJH1Y%3D.

[75] Blanchard C, Gibbs M, Narle G, Brookes C. Learning from communities in the USA and England to promote equity and address the social determinants of health. Glob Health Promot. 2013; 20(4_suppl): 104–112. DOI: 10.1177/1757975913501006 24722749

[76] Haber R, Wong E. Learning from others: comprehensive health equity strategies in Europe; 2013. Accessed January 24, 2022. https://regroup-production.s3.amazonaws.com/documents/ReviewReference/315205836/Haber%2C%20Wong%20-%202013%20-%20Learning%20From%20Others%20Comprehensive%20Health%20Equity%20Strategies%20In%20Europe-annotated.pdf?AWSAccessKeyId=AKIAJBZQODCMKJA4H7DA&Expires=1643175322&Signature=fY5vn5UOyMPmlZJqVSRh5I5RgpE%3D.

[77] Donaldson K, Badger K. Impact innovation: Closing health inequities through design. Published online 2016. DOI: 10.48558/F6G1-X229

[78] Evans RE, Moore G, Movsisyan A, Rehfuess E. How can we adapt complex population health interventions for new contexts? Progressing debates and research priorities. J Epidemiol Community Health (1978). Published online September 27, 2020; jech-2020-214468. DOI: 10.1136/jech-2020-214468 PMC7788480 32981892

[79] Bhattacharyya O, Wu D, Mossman K, et al. Criteria to assess potential reverse innovations: opportunities for shared learning between high- and low-income countries. Global Health. 2017; 13(1): 4. DOI: 10.1186/s12992-016-0225-1 28122623 PMC5264440

[80] Guenther G, Shearer J. Landscape assessment: Bringing health to local communities: Strategies from global health. Published online 2016. Accessed January 25, 2022. www.globaltolocal.org.

[81] Rowthorn V, Plum AJ, Zervos J. Legal and regulatory barriers to reverse innovation. Ann Glob Health. 2017; 82(6): 991. DOI: 10.1016/j.aogh.2016.10.013 28314501

[82] Govindarajan V, Ramamurti R. Five industry-changing principles from India. Published online 2018. DOI: 10.48558/MHNE-J057

[83] Harris M, Dadwal V, Syed SB. Review of the reverse innovation series in globalization and health—where are we and what else is needed? Global Health. 2020; 16(1): 26–30. DOI: 10.1186/s12992-020-00555-6 32216798 PMC7098109

[84] Richard L, Furler J, Densley K, et al. Equity of access to primary healthcare for vulnerable populations: the IMPACT international online survey of innovations. Int J Equity Health. 2016; 15(1): 64. DOI: 10.1186/s12939-016-0351-7 27068028 PMC4828803

[85] Russell G, Kunin M, Harris M, et al. Improving access to primary healthcare for vulnerable populations in Australia and Canada: protocol for a mixed-method evaluation of six complex interventions. BMJ Open. 2019; 9(7): e027869. DOI: 10.1136/bmjopen-2018-027869 PMC6661687 31352414

[86] Pinheiro MC, Strickland M. Social entrepreneurial pathways to a culture of wellbeing; 2016.

[87] Sawin E, McCauley S, Edberg S, Mwaura G, Gutierrez MJ. Multisolving at the intersection of health and climate; 2018. Accessed January 24, 2022. https://www.climateinteractive.org/ci-topics/multisolving/multisolving-at-the-intersection-of-health-and-climate/.

[88] Dearing JW, Lapinski M. Multisolving innovations for climate and health: Message framing to achieve broad public support. Health Aff. 2020; 39(12): 2175–2181. DOI: 10.1377/hlthaff.2020.01170 33284709

[89] Mendel P, Davis L, Turner S, et al. Co-design of services for health and reentry (CO-SHARE): An experience-based co-design (EBCD) pilot study with individuals returning to community from jail and service providers in Los Angeles County. RAND Corporation; 2019. DOI: 10.7249/RR2844

[90] Chapman R, Osman M, Raige H, Mohamed S, Egal N. Seeking Mama Amaan (Safe Motherhood) in Seattle during COVID-19. Health Alliance International. Published July 13, 2020. Accessed January 25, 2022. https://www.healthallianceinternational.org/seeking-mama-amaan-during-covid-19/.

[91] Plum A. Reverse innovation in Healthcare: SDGs bring global health to Detroit; 2017. http://survey.hshsl.umaryland.edu/?url=https://search.ebscohost.com/login.aspx?direct=true&db=ir00217a&AN=umda.10713.7994&site=eds-live.

[92] Loewenson R, Beznec P, Coelho V, et al. Building social power and participation in local health systems: Learning from practice. Published online January 2017. DOI: 10.13140/RG.2.2.18191.48806

[93] Prentiss T, Tamler I, Plum A. et al. Community health workers as innovators: Methods and results from a tele-education pilot for community health workers in Detroit, Michigan; 2017. DOI: 10.24251/HICSS.2017.396

[94] Wagner B. South Valley community partnerships for health equity applying lessons earned in Cuba. https://www.groundworksnm.org/news/south-valley-community-partnership-health-equity-applying-lessons-learned-cuba/2016-1-21.

[95] Behforouz HL, Farmer PE, Mukherjee JS. From directly observed therapy to accompagnateurs: Enhancing AIDS treatment outcomes in Haiti and in Boston. Clinical Infectious Diseases. 2004; 38(Supplement_5): S429–S436. DOI: 10.1086/421408 15156434

[96] Taylor A, Siddiqui F. Bringing global health home: The case of global to local in King County, Washington. Ann Glob Health. 2017; 82(6): 972. DOI: 10.1016/j.aogh.2016.11.006 28314499

[97] Sugarman J, Reed A. Global learning for US primary health care: A resource & implementation guide. Published online 2021. Accessed January 26, 2022. https://www.globaltolocal.org/wp/wp-content/uploads/2021/10/Global-Learning-for-US-PHC.pdf.

[98] Dearing JW, Singhal A. New directions for diffusion of innovations research: Dissemination, implementation, and positive deviance. Hum Behav Emerg Technol. 2020; 2(4): 307–313. DOI: 10.1002/hbe2.216

[99] Brown TM, Cueto M, Fee E. The World Health Organization and the transition from ‘international’ to ‘global’ public health. Am J Public Health. 2006; 96(1): 62–72. DOI: 10.2105/AJPH.2004.050831 16322464 PMC1470434

